# Caspase-dependent apoptosis induces reactivation and gliogenesis of astrocytes in adult mice

**DOI:** 10.3389/fncel.2022.1054956

**Published:** 2022-11-30

**Authors:** Seung-Chan Kim, Jae-Yong Park, Eun Mi Hwang

**Affiliations:** ^1^Brain Science Institute, Korea Institute of Science and Technology, Seoul, South Korea; ^2^Department of Integrated Biomedical and Life Science Graduate School, Korea University, Seoul, South Korea; ^3^Division of Bio-Medical Science and Technology, KIST School, Korea University of Science and Technology (UST), Seoul, South Korea

**Keywords:** astrocyte, gliogenesis, caspase-3, transgenic mice, memory

## Abstract

Astrocytes play an important role in increasing synaptic plasticity, regulating endogenous homeostasis, and contributing to neuroprotection but become overactivated or apoptotic in persistent neuroinflammatory responses or pathological conditions. Although gliogenesis under these conditions may be essential for neuronal protection, much remains unknown. Here, we generated new conditional transgenic mice (cTg) that can induce apoptosis via Cre-dependent active caspase-3 (taCasp3-2A-TEVp) without pathological conditions. We induced apoptosis of hippocampal CA1 astrocytes in cTg mice using GFAP promoter-driven adeno-associated virus (AAV) containing Cre recombinase. Activated caspase-3 was detected in astrocytes of the hippocampal CA1, and the number of astrocytes decreased sharply at 1 week but recovered at 2 weeks and was maintained until 4 weeks. Nuclear factor 1A (NF1A) mRNA, an important transcription factor for hippocampal reactive astrocytes, was significantly increased only at week 1. Interestingly, all reactive markers (pan, A1, A2) increased despite the decreased number of astrocytes at week 1, and there was no change in monoamine oxidase B (MAOB) observed in astrocytes of animal models of degenerative brain disease. Extensive CA1 astrocyte depletion at week 1 induced cognitive deficits; however, both recovered at weeks 2 and 4. Overall, transient hippocampal astrocyte depletion caused by apoptosis restored cell number and function within 2 weeks and did not induce significant neurotoxicity. Therefore, cTg mice are valuable as an in vivo animal model for studying gliogenesis in multiple regions of the adult brain.

## Introduction

Astrocytes are the predominant type of glial cell in the brain, and their dysfunction is believed to be responsible for the pathogenesis of neurodevelopmental and neurodegenerative disorders (Liddelow et al., 2017). Adult astrocytes reactivated by various aetiologies either replace dead astrocytes through proliferation and migration or create glia-scars to isolate the damaged area from the surrounding area (Hara et al., 2017; Liddelow and Barres, 2017). Recently, it has been shown that severely reactive astrocytes exhibit neurotoxicity by generating H_2_O_2_ through the monoamine oxidase B (MAOB) is an enzyme involved in the production of GABA in astrocytes. GABA released from reactive astrocytes affects nearby neurons, and increased enzyme activity of MAOB has been reported in neurodegenerative disease models such as Parkinson’s disease (Mallajosyula et al., 2008) and Alzheimer’s disease (Chun et al., 2020; Ju et al., 2022). There may be steps in the process of astrocyte reactivation, and follow-up studies are needed to classify these steps (Escartin et al., 2021). Currently, reactive astrocytes can be classified into two phenotypes according to their distinct functions: A1 and A2 (Liddelow et al., 2017). A1 astrocytes are neurotoxic, as an underlying pathological response of the central nervous system to infection, injury, and various neurodegenerative disorders. In contrast, A2 astrocytes are induced by ischemia, which promotes neuronal repair and survival. In fact, many subsequent studies have shown that inflammatory responses caused by disease and aging promote the activation of A1 astrocytes (Zhang et al., 2016; Clarke et al., 2018; Xu et al., 2018; Qian et al., 2019; Zou et al., 2019; Li et al., 2020), and that A2 astrocytes are involved in the proliferation of astrocytes (Liddelow and Barres, 2017). However, recent single-cell-level studies have suggested that astrocytes are heterogeneous and can be further subdivided and classified according to brain regions and stimuli-specific reactivity (Miller, 2018; Wang et al., 2020; Hasel et al., 2021; Sadick et al., 2022; Spurgat and Tang, 2022). Gliogenesis occurs from late neural stem cells (NSCs) during development, but in adults, it can also occur by the proliferation of reactive astrocytes (Wanner et al., 2013). Nuclear factor 1A (NF1A) has been identified as an important transcription factor for the generation and differentiation of astrocytes, and NF1A deficiency prevents astrocyte-neuron communication (Deneen et al., 2006; Laug et al., 2019).

Here, we report the generation and characterisation of activated caspase-3 conditional transgenic [taCasp3-Tobacco Etch Virus protease (TEVp)-cTg] mice. These mice can be used to induce apoptosis by overexpressing active caspase-3 in a Cre-dependent, cell type-specific manner. In this study, we induced astrocyte-specific apoptosis in the hippocampus of cTg mice and confirmed that these mice worked very effectively. We also confirmed that various known astrocyte markers change with time after the expression of active caspase-3 and investigated whether astrocyte apoptosis affects neuronal function through behavioural experiments. Our data suggest that these cTg mice can be used as a valuable model to study the reactivation and gliogenesis of astrocytes that show heterogeneity in the brain region in adults.

## Materials and methods

### Animal

All experiments were with 8–12 weeks old male mice. Animal cares were conducted in accordance with the institutional guidelines of the Korea Institute of Science and Technology (KIST IACUC).

### Generation of taCasp3-2A-TEVp cTg mice

The gBlocks plasmid fragments (Integrated DNA Technologies, Coralville, USA) containing the FseI-taCasp3-2A-TEVp-FseI cassette were inserted into the same restriction enzyme site of the Ai6 plasmid vector to generate conditional Caspase3 transgene constructs. Next, the Caspase3-2A-TEVp (Tobacco Etch Virus protease) transgene plasmid was double-cut using the AvrII/SacI site and microinjected into embryonic stem cells to generate cTG mice (Macrogen, Seoul, South Korea).

### Genotyping polymerase chain reaction

The cTg mice were genotyped using the following primer pair: 5′-CAG AGG GGA TCG TTG TAG AAG-3 (forward), 5′-TGC TGG AGA TCG GGT TGT AGT-3 (reverse).

### Primary astrocytes culture

Primary astrocytes were cultured from the cortical region of P1 pups as described previously (Schildge et al., 2013). Primary astrocytes were grown in DMEM supplemented with 10% fetal bovine serum and 1% penicillin/streptomycin at 37°C in a 5% CO2 incubator. After 3 days, the culture medium was changed to wash astrocytes and eliminate cell debris. AAV vector constructs were electroporated into primary cultured astrocytes on day 7 using a NEON kit (Invitrogen, Carlsbad, CA, USA) according to the manufacturer’s instructions.

### Stereotaxic injection

The taCasp3-2A-TEVp cTg mice were anaesthetised by an intraperitoneal injection of avertin (2,2,2-tribromoethanol in 2-methyl butanol). cTg mice were bead-fixed in a stereotaxic apparatus. 0.5 μl of AAV5-GFAPp- Hemagglutinin (HA)-Blue fluorescent protein (BFP) or BFP-Cre virus was injected in the hippocampus (AP: –1.9 mm, ML: ± 1.4 mm, DV: –1.6 mm from bregma) at 0.1 nl/min.

### Immunofluorescence staining

Mouse brains were isolated following perfusion surgery with saline and 4% paraformaldehyde in PBS and 40 μm-thick tissue sections were sliced using a Leica vibratome. Hippocampal tissues were permeabilised with 0.5% Triton X-100 in PBS for 30 min. The sections were then blocked with 3% bovine serum albumin (BSA) and 5% donkey serum in 0.3% Triton X-100 in PBS for 1 h. The blocked tissues were incubated with primary antibodies in blocking solution at 4°C overnight. The following primary antibodies were used in these studies: mouse anti-NEUN (MERCK, MAB377, 1:500), chicken anti-GFAP (Thermo Fisher, #PA1-10004, 1:500), rabbit anti-cleaved Caspase-3 (Cell signalling, #9661, 1:500), rat anti-Ki67 (Invitrogen, #14569882, 1:500), and rat anti-HA (Roche, #11867423001, 1:500). For detection, Alexa fluorescence-tagged secondary antibodies were used following the addition of 488-, 594- conjugated secondary antibody (Jackson ImmunoResearch Laboratories, 1:300) and incubated for 1 h at RT. After incubation and washing, the slides were mounted using DAKO mounting solution (Agilent, Steven Creek Blvd, Santa Clara, USA). The mounted samples were observed using a Nikon A1 confocal microscope.

### Western blotting

Cell lysates were extracted using RIPA buffer (Thermo Fisher, #89900) containing a protease inhibitor cocktail. Lysates of total proteins were separated by SDS-PAGE on 10% gels and transferred to a PVDF membrane. The membranes were blocked with 5% skim milk for 1 h at room temperature. The blots were incubated with primary antibodies at 4°C overnight. The primary antibodies used were rabbit anti-cleaved Caspase-3 (Cell signalling, #9661, 1:1,000; Cell Signalling Technology), mouse anti-actin (A5411, 1:1,000; Sigma Aldrich), and rat anti-HA (#11867423001, 1:1,000; Roche). The membranes were then incubated with horseradish peroxidase-conjugated secondary antibodies. Blots were washed, and immunoreactivity was detected using enhanced chemiluminescence.

### Quantitative real-time polymerase chain reaction

RiboEx reagent (GeneAll, Seoul, South Korea) was used to extract total RNA from hippocampal tissues of cTg mice. Total RNA was isolated using an RNA Purification Kit (GeneAll, Seoul, South Korea). SensiFAST™ cDNA Synthesis Kit (Bioline, London, UK) according to the manufacturer’s instructions. The qPCR assay was performed according to the manufacturer’s instructions using the SensiFAST™ HI-ROX kit (Bioline, London, UK), followed by detection using Applied Biosystems (Foster City, CA, USA). Primers and probes were purchased from IDT (Integrated DNA Technologies Pte. Ltd., Singapore), and sequence information was included in the Supplementary material. GAPDH was used as an internal normalization control. All experiments were performed in triplicates. The 2^–Δ^ ^ΔCT^ method was used to calculate the relative gene expression (Livak and Schmittgen, 2001).

### Behaviour test

To assess hippocampal function, we performed four behavioural tests. Before starting each experiment, mice were habituated for 1 h.

#### Spontaneous alternation y-maze test

The Y-maze test was performed as described in previously (Miedel et al., 2017). Spatial working memory was assessed by measuring spontaneous alternations in the Y-maze apparatus. Initially, mice were placed within the central zone of the maze, and the mice was allowed to freely explore all three arms (A, B and C) for 10 min in Y-maze. Spontaneous alternation occurs when mice enter a different arm in three consecutive arm entries (ABC, ACB, or CBA, but not the ACA). The percentage of spontaneous alternation was calculated using the following formula: % alternation = (number of spontaneous alternations/total number of arm entries-2) × 100.

#### Novel object recognition test

Animals were first exposed to an open field test for 10 min before being exposed to the same two objects for 10 min during a training session. In the testing session, mice were placed in the same open field in which one of the familiar objects was replaced with a new one and recorded for 10 min.

#### Open field test

To evaluate the effects of apoptotic astrocyte induced by active caspase-3 on exploratory locomotor activity, mice were tested 1, 2, and 4 weeks after AAV injection. The open field test was performed according to previous methods (Kim et al., 2019), using an open field maze to measure locomotor activity and anxiety level as time spent in the centre zone of the maze.

#### Elevated plus maze test

The EPM is a plus-shaped apparatus that consists of two opposite open arms and two opposite closed arms. The animals were tested according to a previous study (Ari et al., 2019), to measure the time spent in each arm for 5 min.

### Cell counting and mean fluorescence intensity

For astrocytes and proliferating cells, conventional counts and intensity of GFAP (green)/Ki67 (red)-positive cells from confocal images were evaluated using the ImageJ free software program.

### Sholl analysis

Sholl analysis was performed as previously described to measure the changes in astrocyte morphology (Sullivan et al., 2010). The process intersection of astrocytes was investigated according to the sholl analysis plugin using Fiji-free software program^1^ from confocal Z-stack images using a Nikon A1 microscope.

### Statistical analysis

All data are expressed as mean ± standard error of the mean (S.E.M). Data were analysed using two-way ANOVA followed by Tukey’s correction using GraphPad Prism software version 9.0. Differences were considered statistically significant at *p* < 0.05.

## Results

### Generation and validation of conditional active caspase-3 transgenic mice

**FIGURE 1:**
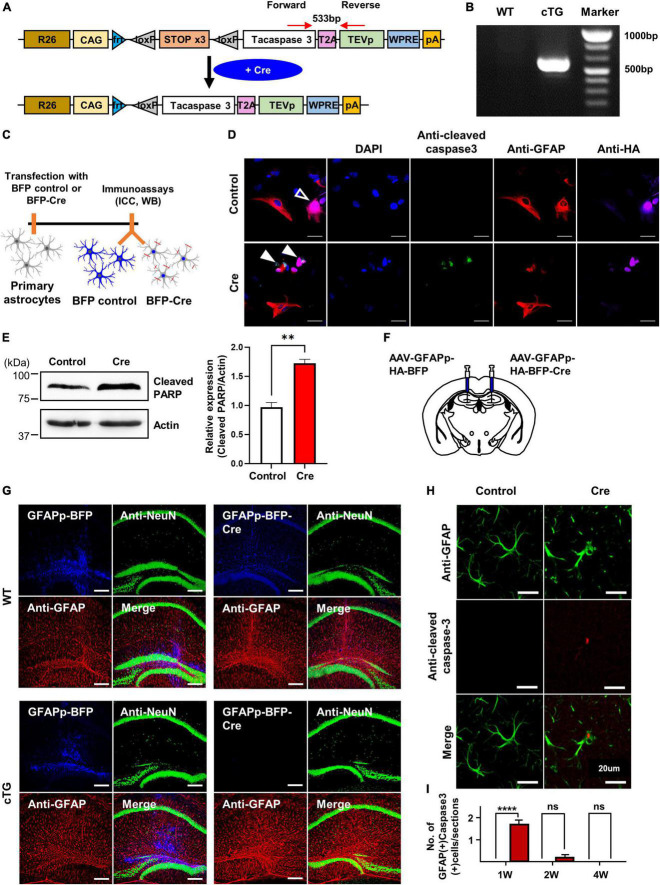
The generation and validation of active casapse-3 cTg mice. **(A)** A schematic design of Caspase-3 cTg mice. Inserted genes were designed to express activated caspase-3 with the CAG strong promoter expressed by Cre. **(B)** Genotype PCR results with designed forward and reverse primer pair. **(C)** A diagram showing the timeline of in vitro experiment. Cultured primary astrocytes from Caspase-3 cTg mice were electroporated with BFP-control or BFP-Cre construct using NEON kit. **(D)** Microscopic imaging data indicating the co-localization of GFAP and HA without cleaved caspase-3 in the control group (empty arrow) and co-localization of HA and cleaved caspase-3 without GFAP in Cre group (filled arrow). **(E)** Western blotting data showing the expression of cleaved PARP, known as downstream of caspase-3 pathway. The amount of cleaved PARP increased by Cre expression. **(F)** A schematic design showing the injection of AAV-virus containing GFAP promoter, a selective promoter of astrocyte. **(G)** Microscopic data with no difference in GFAP and NeuN signals between control and Cre virus group in WT mice (upper) and showing the disappearance of viral signal by Cre virus in cTg mice (lower). **(H)** Microscopic imaging data showing the GFAP without cleaved caspase-3 in the control group and simultaneously an expression of GFAP and cleaved caspase-3 in Cre group. **(I)** Quantitative data on the number of GFAP-positive and active caspase-3 positive cells from control or Cre group (# of sections = 10).

To create conditional mice that induce cell-autonomous apoptosis, we designed a vector (loxP-flanked STOP cassette-taCasp3-2A-TEVp-polyA) overexpressing active caspase-3 using Cre and used it to generate transgenic mice (Figure 1A). The caspase-3 cTg mice obtained were validated by genotyping PCR using the designed forward and reverse primer pairs (Figure 1B). We determined whether apoptosis was induced in these transgenic mice by Cre-dependent recombination through immunocytochemistry and western blotting experiments in cultured primary astrocytes (Figure 1C). Using confocal microscopy, we first confirmed that cleaved caspase-3 was selectively expressed in Cre-expressing cells (HA-positive cells; Figure 1D). We tried to confirm the expression of cleaved PARP, known as apoptosis marker, to investigate the signal following activated caspase-3 (Agarwal et al., 2009). We also confirmed CRE-mediated apoptosis by western blot analysis of increased cleaved PARP (cPARP; Poly-ADP-ribse polymerase) expression known as downstream of the caspase-3 signalling pathway (Figure 1E). To verify Cre-induced apoptosis in vivo, we injected astrocyte-specific AAV containing Cre (AAV-GFAPp-HA-BFP-Cre) into the hippocampal region of the brain and performed immunohistochemical experiments 2 weeks later (Figures 1F,G). We expected that the number of GFAP-positive cells (astrocytes) would decrease because astrocytes effectively undergo apoptosis due to Cre expression, but surprisingly, no difference was found. Therefore, we checked whether NeuN-positive cells (neurons) were significantly reduced 2 weeks after injection of neuron-specific Cre-AAV (AAV-hSyn-HA-BFP-Cre) into the hippocampus (Supplementary Figure 1A). It was observed that neurones were effectively removed by Cre and cleaved caspase-3 was expressed at the injection site (Supplementary Figures 1B–D). Similarly, some expression of cleaved caspase-3 was also observed at the site of astrocyte-specific Cre-AAV injection compared to control AAV (Figures 1H,I). Taken together, we demonstrated that the newly generated active caspase-3 cTg mice worked effectively in a Cre-dependent manner both in cultured cells and in vivo.

### Astrocytes mostly recovered within 2 weeks from apoptosis

**FIGURE 2:**
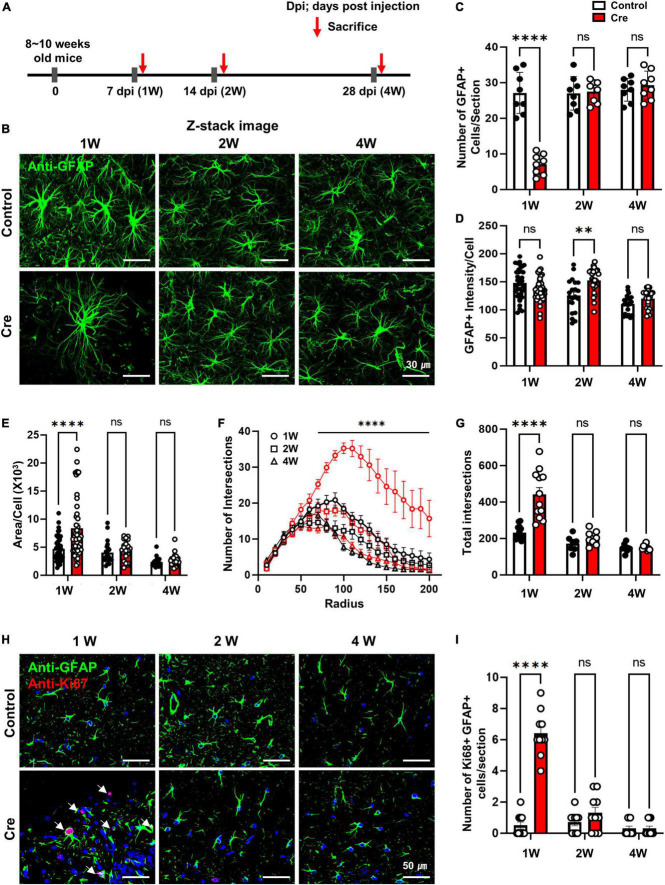
Induction of apoptosis with Cre in hippocampal astrocytes of active caspase-3 cTg mice. **(A)** A diagram showing the timeline of experimentation. **(B)** Following the administration of AAV-GFAPp-HA-BFP-Control or HA-BFP-Cre virus, slice tissue of brain in cTg mice were performed to immunostaining to analyze the expression of GFAP-positive astrocytes; Anti-GFAP (green); Z-stack imaging. **(C)** The number of GFAP-positive cells per section (# of sections = 8). (**D,E**) Analyze the area and intensity of GFAP-positive cells per single cell (1W; control *n* = 46, Cre *n* = 45, 2W; control *n* = 21, Cre *n* = 24, 4W; control *n* = 21, Cre *n* = 24). **(F)** The number of intersections per radius (in 200 μm) using the sholl analysis (1W; control *n* = 14, Cre *n* = 12, 2W; control *n* = 7, Cre *n* = 8, 4W; control *n* = 8, Cre *n* = 9). **(G)** The total number of intersections (in 200 μm) using the sholl analysis (1W; control *n* = 14, Cre *n* = 12, 2W; control *n* = 7, Cre *n* = 8, 4W; control *n* = 8, Cre *n* = 8). **(H)** Following the administration of AAV-GFAPp-HA-BFP-Control or HA-BFP-Cre virus, slice tissue of brain in cTg mice were performed to immunostaining to analyze the expression of Ki67 in GFAP-positive astrocytes; Anti-GFAP (green), anti-Ki67 (red). **(I)** Number of GFAP-positive and Ki67-positive cells per sections (# of sections = 10). Data are represented as the means ± SEM (****p < 0.00001 according to two-way ANOVA and Turkey’s test, ns; not significant).

From the results shown in Figure 1, it was confirmed that Cre-dependent apoptosis of astrocytes recovered to some extent within 2 weeks in active caspase-3 cTg mice, so it is necessary to study the changes in astrocytes at various times in more detail. We investigated the number and reactivity of astrocytes and newly dividing cells in hippocampal tissues 1, 2, and 4 weeks after virus injection (Figure 2A). When we observed the hippocampal tissue at low magnification, the expression of control-AAV gradually increased, and the expression of Cre-AAV could not be detected due to apoptosis (Supplementary Figure 2). Interestingly, extensive deletion of astrocytes in the hippocampus was clearly confirmed 1 week after Cre-AAV injection. Immunofluorescence staining for GFAP showed that the number of astrocytes significantly decreased 1 week after Cre-AAV injection, but the size of the remaining astrocytes increased, and all recovered within 2 weeks (Figures 2B–D). However, GFAP intensity showed a significant increase at 2 weeks, which is thought to be because the reactivity of astrocytes was not completely restored at 2 weeks (Figure 2E). We additionally performed Sholl analysis to precisely confirm the morphological changes in astrocytes, and only significant changes were observed at 1 week in the Cre-AAV injection group (Figures 2F,G). Next, we investigated whether new proliferation occurred using Ki67 staining (red), a marker of proliferating cells near the apoptotic zone (Figure 2H). As a result of analysing the number of Ki67-positive cells, it increased at the week 1, but was hardly detected at the 2 weeks and 4 weeks (Figure 2I). Therefore, these data suggest that astrocytes are effectively eliminated within 1 week by caspase-dependent apoptosis in vivo, which induces rapid cell proliferation and can be mostly recovered within 2 weeks.

### Apoptosis in astrocytes induces reactive astrocytic markers without neurotoxicity

**FIGURE 3:**
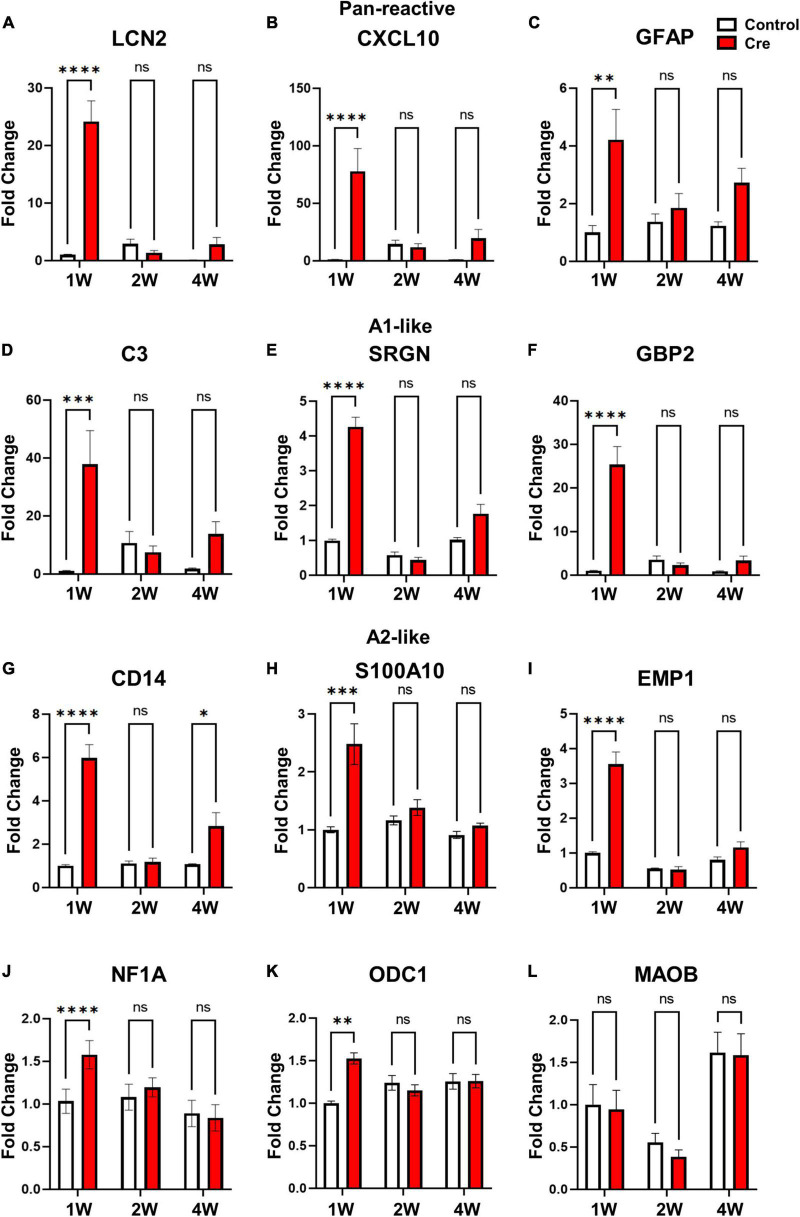
mRNA Expression of various reactive astrocytic markers in hippocampal astrocyte-specific active caspase-3 cTg mice. mRNA expression of markers of reactive astrocytes using qRT-PCR (# of animals; 1W *n* = 4, 2W *n* = 3, 4W *n* = 3, repeat 3 times). **(A–C)** Pan-reactive marker; LCN2, CXCL10, GFAP. **(D–F)** A1-like marker (neurotoxic); C3; complement 3, SRGN; serglycin, GBP2; guanylate-binding protein 2. **(G–I)** A2-like marker **(neuroprotective)**; CD14, S100A10; S100 calcium binding protein A1, EMP1; erythrocyte membrane protein 1. **(J)** Gliogenesis marker; NF1A (Nuclear factor 1A). **(K)** Urea cycle; ODC1 (ornithine decarboxylase). **(L)** Another reactive astrocyte marker; MAOB (monoamine oxidase B). Data are represented as the means ± SEM (*p < 0.05, **p < 0.01, ****p < 0.00001 according to two-way ANOVA and Turkey’s test, ns; not significant).

Reactive astrocytes have been reported to be classified into neurotoxic-A1 type and neuroprotective-A2 type (Liddelow et al., 2017). We determined whether the reactive astrocytes shown in the results in Figure 2 were (1) of which type and (2) neurotoxic. First, we confirmed the expression of various reactive astrocyte markers (Pan, A1, and A2) in the virus injection site tissue at 1, 2, and 4 weeks. Interestingly, mRNA levels of pan-reactive (Lcn2, Cxcl10, GFAP), neurotoxic A1-like (C3; complement 3, SRGN; serglycin, GBP2; guanylate-binding protein 2), and neuroprotective A2-like (CD14, EMP1; erythrocyte membrane protein 1, S100A1; S100 calcium binding protein A1) markers all significantly increased at 1 week only (Figures 3A–I). In addition, we confirmed that the mRNAs levels of NF1A (nuclear factor 1A) and ODC1 (ornithine decarboxylase), which were reported to increase in reactive astrocytes (Laug et al., 2019; Ju et al., 2022), also increased only after 1 week (Figures 3J,K). However, no significant difference was found in the case of MAOB (monoamine oxidase B) mRNA, which is known as the enzyme for GABA production in reactive hippocampal astrocytes (Jo et al., 2014; Figure 3L). Next, because the A1-like markers increased to a higher fold change (FC) than the A2-like markers, we examined whether there was a neurotoxic effect. To confirm the neurotoxic effect, mRNA levels of various neuronal markers (NeuN, MAP2, Gria1, and Grin2a) were investigated by qRT-PCR, and it was confirmed that there was no difference at any time point (Supplementary Figures 3A–D). We additionally checked whether the number of neurons changed at the same time condition, and did not observe a significant change (Supplementary Figures 3E,F). From these data, we suggest that transient apoptotic death of astrocytes increases the overall reactivity of the surrounding astrocytes, but it is reversible and does not induce neurotoxicity.

### Deletion of astrocytes actually impairs the function of hippocampal neuron

**FIGURE 4:**
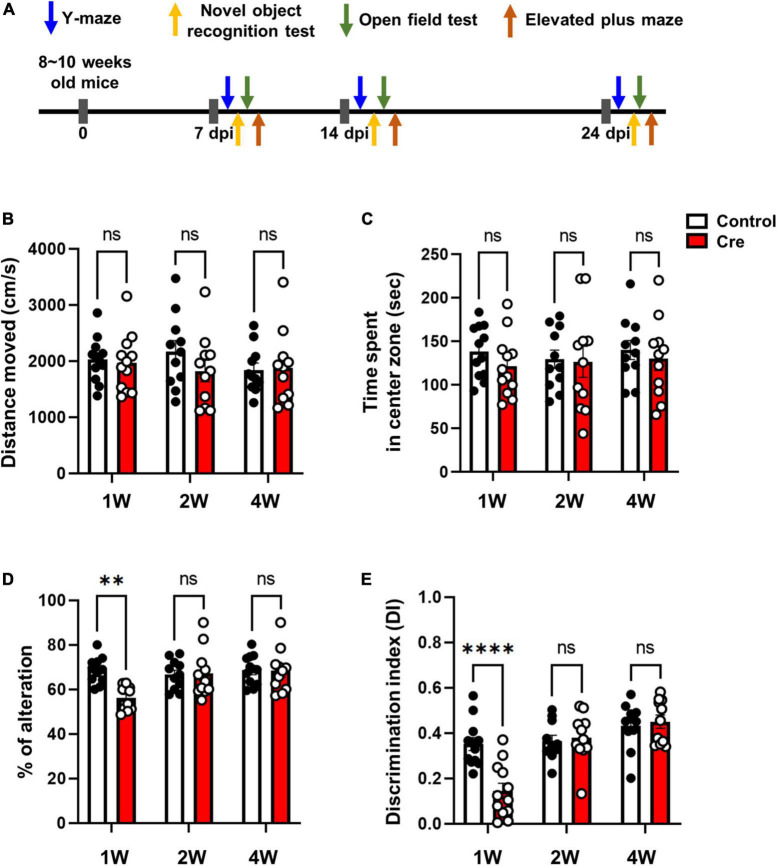
Behavioral tests in hippocampal astrocyte-specific active caspase-3 cTg mice. **(A)** A diagram showing the timeline of behavioral experiments. **(B)** Measurement of total distance traveled in the open field test. **(C)** Measurement of the time spent in the center zone in the open field test. **(D)** Percentage of spontaneous alternation in the Y-maze test. **(E)** Rectification of index in the new object recognition test. Data are represented as the means ± SEM (**p < 0.01 according to two-way ANOVA and Turkey’s test, ns; not significant).

Among several brain regions, the hippocampus plays a critical role in learning, memory and cognition functions (Bird and Burgess, 2008; Lisman et al., 2017), and it is well known that astrocytes interact with neurons through tripartite synapses in this process (Araque et al., 1999). Since we removed astrocytes without nerve damage 1 week after virus injection using active cTg mice (Figures 2, 3), we investigated the effect of ablation of neuron-astrocyte interactions on memory and cognition (Figure 4A). First, we performed an open field test and elevated plus maze test to identify motor- and anxiety-like behaviours. As a result of the total distance travelled in the open field test, there was no difference in locomotion activity (Figure 4B). There was no difference in the time spent in the central zone of the open field. (Figure 4C). In addition, there was no difference in arm opening and closing times in the elevated plus maze test; therefore, it did not affect anxiety levels (Supplementary Figures 4A,B). Next, we performed a Y-maze test to confirm spatial working memory, and a novel object recognition test to confirm learning and memory. We observed a significant reduction in spontaneous alternation in the Y-maze test of mice injected with Cre at week 1 (Figure 4D). In addition, novel object recognition tests are used to estimate cognitive memory and are based on the natural tendency to explore new objects longer than familiar ones (Cohen and Stackman, 2015). Active caspase-3 cTg mice injected with Cre virus had a lower tendency to interact with new individuals than control mice (Figure 4E). These results indicate that the removal of astrocytes from the hippocampus can induce learning and memory dysfunction.

## Discussion

In various pathological brain environments, astrocytes can become reactive glial cells or die via apoptosis. Indeed, there are recent reports of astrocyte apoptosis by lipopolysaccharide (LPS) or other inflammatory stimuli that are widely used to mimic systemic inflammatory conditions (Suk et al., 2001; Sun et al., 2016). To understand the importance of the physiological role of astrocytes, it is necessary to examine their role in brain function after selectively removing astrocytes.

In this study, we generated novel conditionally transgenic (cTg) mice expressing Cre-dependent active caspase-3 (Figure 1). Previously, another research group conducted a study to induce astrocyte cell death using the diphtheria toxin receptor and reported that astrocytes did not die even when diphtheria toxin was administered, and the autophagy signalling pathway was activated to increase reactivity (Chun et al., 2020). However, our cTg mice have proven to be a suitable mouse model for inducing cell-autonomous apoptosis in astrocytes, as they express active caspase-3 in a Cre-dependent manner (Figure 2). Examination of the apoptotic effect of astrocytes by excluding the influence of surrounding cells (microglia or neurons) using this cTg mouse revealed that the deficiency due to apoptosis of astrocytes rapidly recovered within 2 weeks by gliogenesis. Interestingly, the expression of A1-like (neurotoxic) markers, as well as A2-like (neuroprotective) markers speculated to be required for gliogenesis, also increased at 1 week, and no neurotoxic phenotype was found. We could not determine whether the increase in A1-like markers was due to cell proliferation or death. However, the expression of the A1-like marker did not directly induce neurotoxicity in vivo, at least for a short period of time. In addition, we confirmed that the expression of NF1A and ODC1, which has been reported to be increased in reactive astrocytes (Laug et al., 2019; Ju et al., 2022), also increased. However, there was no difference in the expression of MAOB, which has been reported to be increased in reactive astrocytes in Alzheimer’s disease (Jo et al., 2014), a chronic inflammatory disease (Figure 3). In addition, it is known that gliotransmitters such as D-serine, glutamate, GABA, ATP and BDNF, released from astrocytes, play a very important role in hippocampal spatial memory (Hassanpoor et al., 2014). There was no study showing the effect of inhibiting the release of all these substances by the temporary deletion of only astrocytes. In fact, the function of hippocampal neurons related to memory at 1 week without astrocytes was incomplete in Y-maze and NORT experiments, but was fully recovered at 2 weeks (Figure 4).

Astrocytes, similar to neurons, are heterogeneous in that they can be responsible for different shapes and functions in each area of the brain (Miller, 2018; Spurgat and Tang, 2022). Although we investigated the function of astrocytes in the hippocampal region in this study, it is possible to study the role of astrocytes in various brain regions using cTg mice. Furthermore, based on additional single-cell-level studies, mating with cell type-selective Cre-line mice can further study the role of specific astrocytes. In addition, when using an induced Cre line mouse, it is possible to exclude reactivity according to virus injection in astrocyte studies, which has the advantage of being able to study the apoptosis effect at an early stage (within 1 week). We propose that our novel cTg mice have the potential to be extended to functional studies of various cell types, and can be utilised in the field of research on the reactivation and gliogenesis processes of astrocytes in the brain.

## Data availability statement

The original contributions presented in this study are included in the article/Supplementary material, further inquiries can be directed to the corresponding author.

## Ethics statement

This animal study was reviewed and approved by KIST Institutional Animal Care and Use Committee (IACUC). Written informed consent was obtained from the owners for the participation of their animals in this study.

## Author contributions

EH and J-YP contributed to the study conception and design. S-CK performed all the experiments and wrote the first draft of the manuscript. EH helped to prepare the manuscript. All authors have read and approved the submitted version of the manuscript.

## Footnotes

1
https://imagej.net/software/fiji/downloads
