# Supplementary material for: Caspase-dependent apoptosis induces reactivation and gliogenesis of astrocytes in adult mice
Source: Front Cell Neurosci. 2022 Nov 30;16:1054956. doi: 10.3389/fncel.2022.1054956 (PMC9749822; doi:10.3389/fncel.2022.1054956)
Supplement: Supplementary file 1 [file Data_Sheet_1.PDF]

Supplementary Figure 1

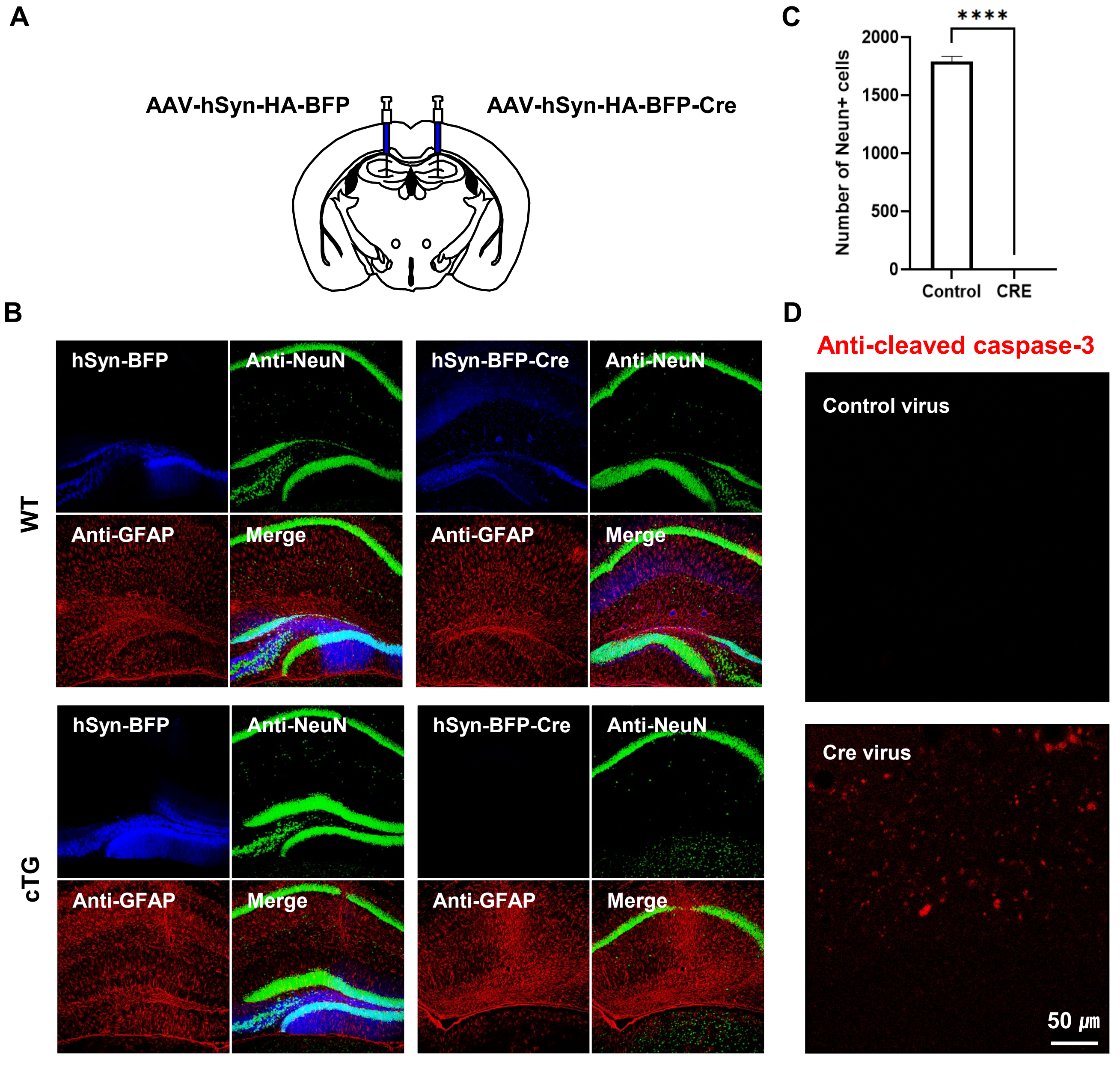

Supplementary Figure 2

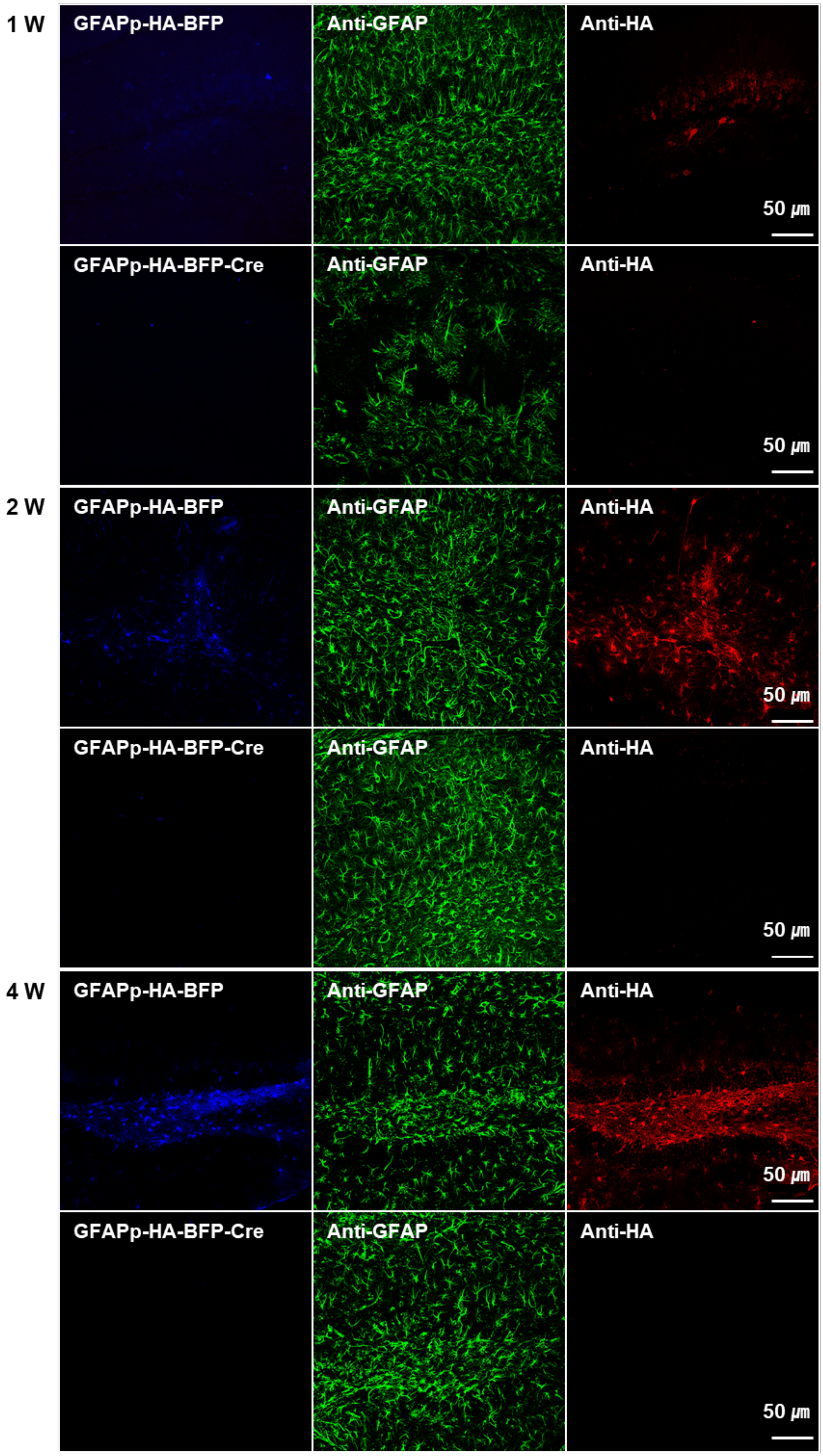

Supplementary Figure 3

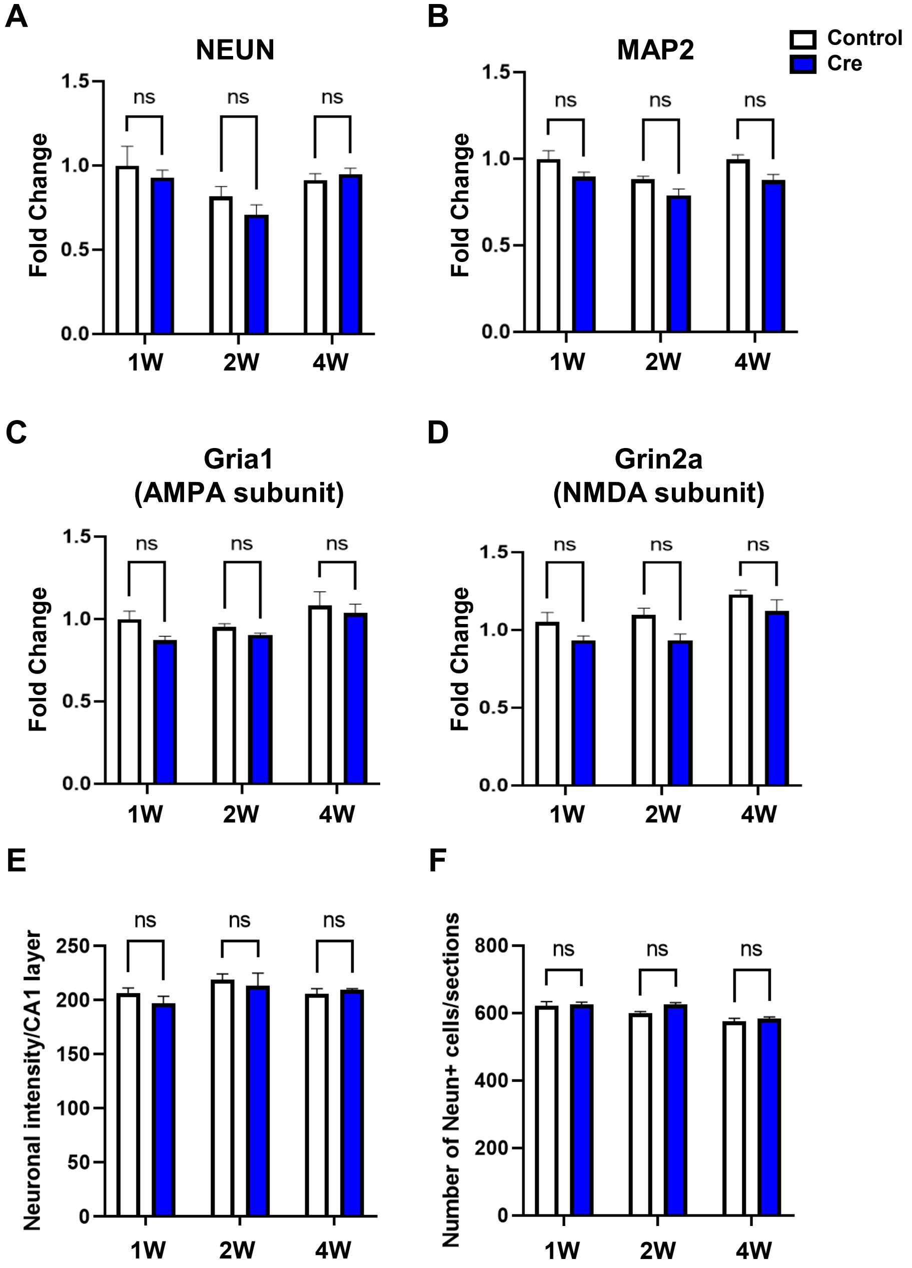

Supplementary Figure 4

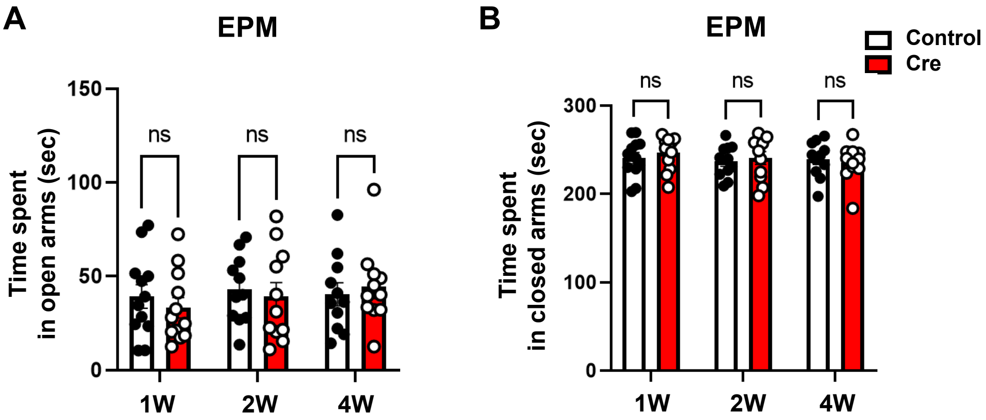

Table 1. Real-time PCR probes and primers

Primer’s probe	Sequence (5’-3’)	Ref Seq No.	Assay ID
LCN2 forward	CTACAATGTCACCTCCATCCTG		
LCN2 reverse	CCTGTGCATATTTCCCAGAGT	NM_008491	Mm.PT.58.10167155
LCN2 probe	56-FAM/TGTTCTGAT/ZEN/CCAGTAGCGACAGCC		
CXCL10 forward	ATTTTCTGCCTCATCCTGCT		
CXCL10 reverse	TGATTTCAAGCTTCCCTATGGC	NM_021274	Mm.PT.58.43575827
CXCL10 probe	56-FAM/ATCCCTCTC/ZEN/GCAAGGACGGTC		
GFAP forward	AACCGCATCACCATTCCTG		
GFAP reverse	GCATCTCCACAGTCTTTACCA	NM_010277	Mm.PT.58.31297710
GFAP probe	56-FAM/CAACCTCCA/ZEN/GATCCGAGAAACCAGC		
C3 forward	CCTTCCACCTTTTTCCTTCACT		
C3 reverse	CTCCAGCCGTAGGACATTG	NM_009778	Mm.PT.58.17325540
C3 probe	56-FAM/AGGGTCCCA/ZEN/GCTACTAGTGCTACTG		
SRGN forward	GCCTTCGTCCTGGTTTGG		
SRGN reverse	CCTCGATGCAGTTCGCAA	NM_011157	Mm.PT.58.41483771
SRGN probe	56-FAM/TTCAGTGCA/ZEN/AGGTTATCCTGCTCGG		
GBP2 forward	GCAGAATTCACCTCATACATCTTG		
GBP2 reverse	GATGGCACCAACATAGGTCTG	NM_010260	Mm.PT.58.1360146
GBP2 probe	56-FAM/CAGGCTCTT/ZEN/TAGACGTGGCCCATT		
CD14 forward	AACTTTCAGAATCTACCGACCA		
CD14 reverse	CAATCTGGCTTCGGATCTGAG	NM_009841	Mm.PT.58.871572.g
CD14 probe	56-FAM/CACCAGAGC/ZEN/CCTGCGAGCTAG		
CD109 forward	CCCACTGTGAGAGACTACAAAG		
CD109 reverse	GAGGTAACATGAGGACAGCTTC	NM_153098	Mm.PT.58.6710335
CD109 probe	56-FAM/ATGAACCAA/ZEN/GGAGACAGGCAGTGC		
EMP1 forward	GCCATTATGCTGTTTGTCTCC		
EMP1 reverse	ATCTTCATTGCCGTAGGACAG	NM_010128	Mm.PT.58.5886962
EMP1 probe	56-FAM/TGCGTAATC/ZEN/TGCAACCATCCAGACG		
MAOB forward	CATGAGGTCTCTGCACTATGG		
MAOB reverse	ACTCACTTGACCAGATCCAC	NM_172778	Mm.PT.58.33530177
MAOB probe	56-FAM/CCTCCACAC/ZEN/TGCTTCACATACCACA		
NFIA forward	CAGTGGATGGCATGAAGTAGA		
NFIA reverse	GCTGGGTGTGAGAAGTAAGG	NM_010905	Mm.PT.58.5716963
NFIA probe	56-FAM/TTGGAAAGT/ZEN/GGAGAGTCGATGGCG		
RBOFX3 forward	ATACATTTCGAGCTGCACCA		
RBFOX3 reverse	CCTCCATAAATCTCAGCACCA	NM_001039168	Mm.PT.58.32889417
RbFOX3 probe	56-FAM/CCATCCTGA/ZEN/TACACGACCGCTCCATA		
MTAP2 forward	AACAGCTAATCTGCCACCTTC		
MTAP2 reverse	GTGACTTTATCCTTCGCCTGT	NM_008632	Mm.PT.58.10819514
MTAP2 probe	56-FAM/CCACTTGCT/ZEN/GCTTCCTCCACTGT		
GRIA1 forward	ATCGAGTTCTGCTACAAATCCC		
GRIA1 reverse	TCCGTATGGCTTCATTGATGG	NM_008165	Mm.PT.58.9021489
GRIA1 probe	56-FAM/AACAGAAAC/ZEN/CCTTCATCCGCTTCGA		
GRIN2A forward	GATTGACCTCGCTCTGCTC		
GRIN2A reverse	TGCTCATCACCTCATTCTTCTC	NM_008170	Mm.PT.58.13771721
GRIN2A probe	56-FAM/CTCTTCCAT/ZEN/CTCACCGTCACCAACA		
