# Supplementary figures and images for: Caspase-dependent apoptosis induces reactivation and gliogenesis of astrocytes in adult mice
Source: Front Cell Neurosci. 2022 Nov 30;16:1054956. doi: 10.3389/fncel.2022.1054956 (PMC9749822; doi:10.3389/fncel.2022.1054956)

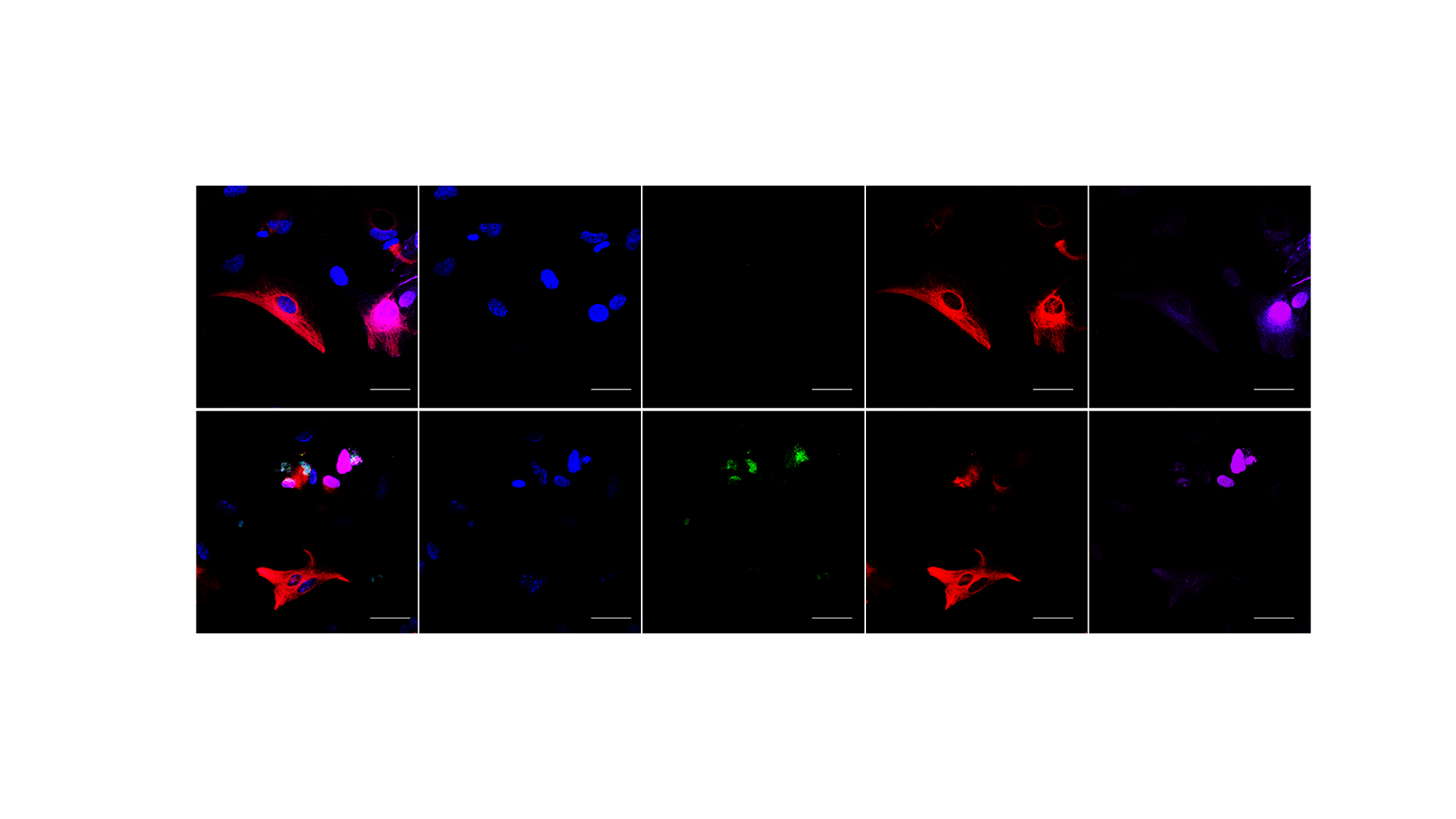

Supplement: Supplementary file 3 [file Image_1.TIF]

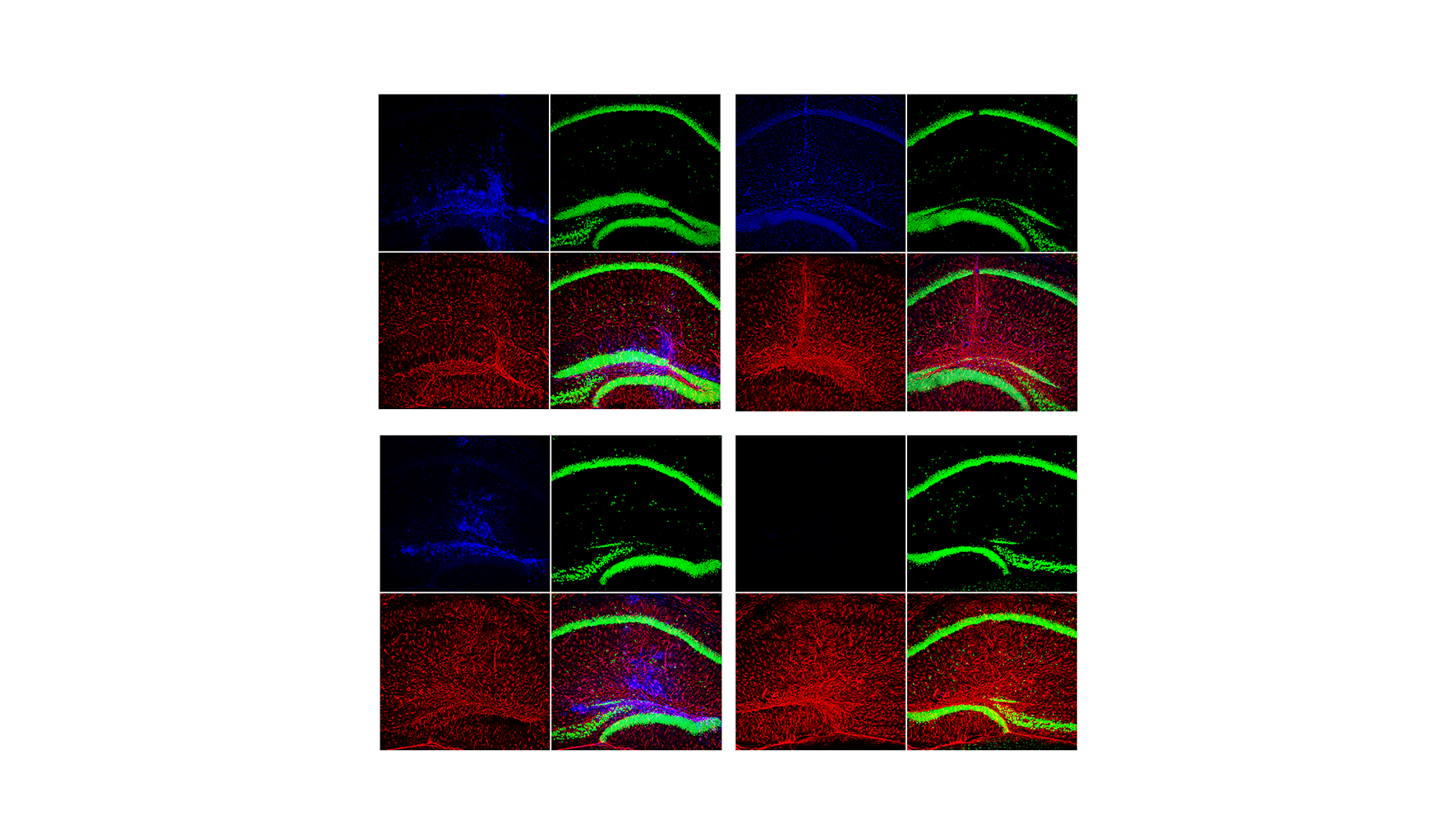

Supplement: Supplementary file 4 [file Image_2.TIF]

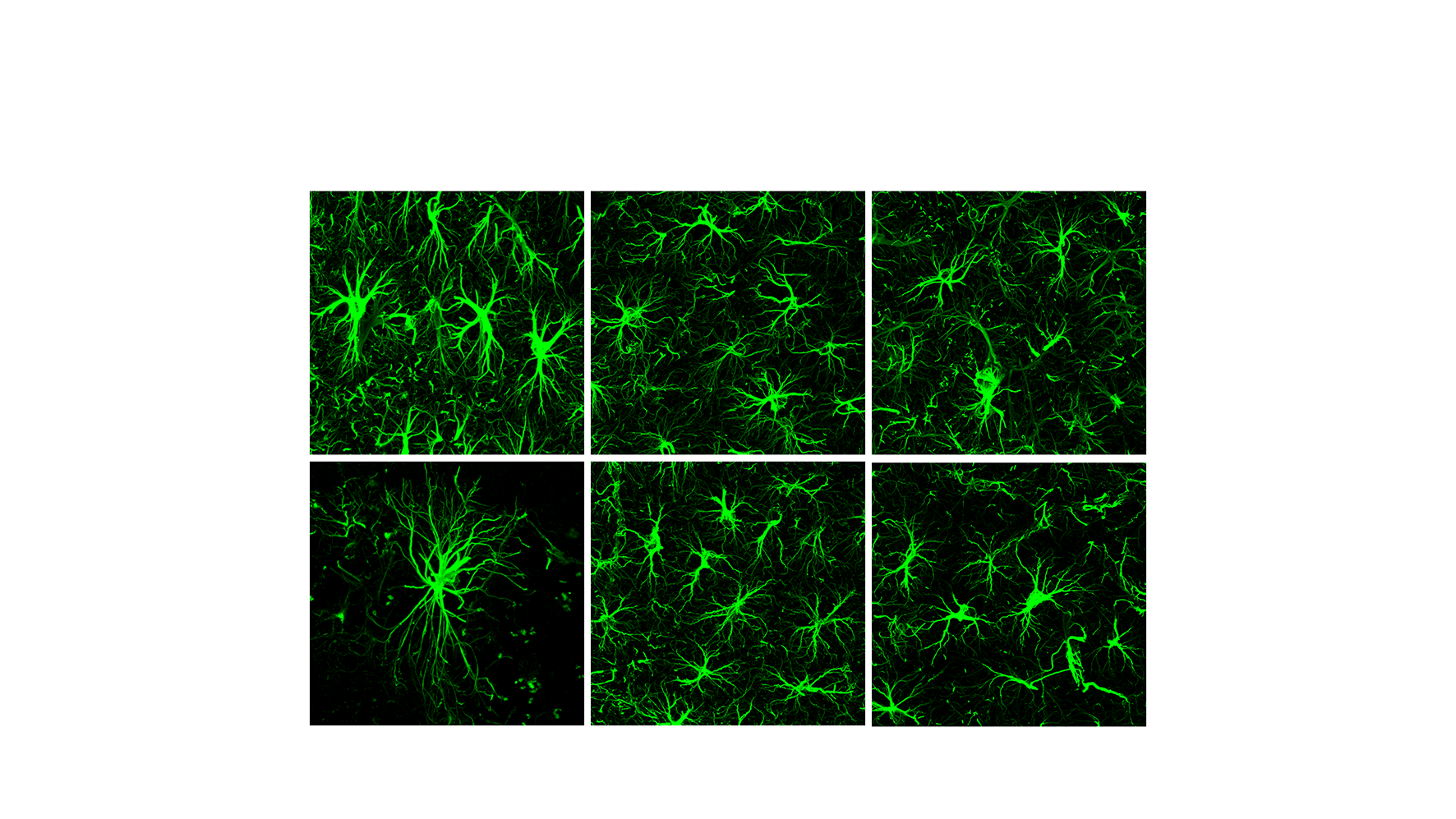

Supplement: Supplementary file 5 [file Image_3.TIF]

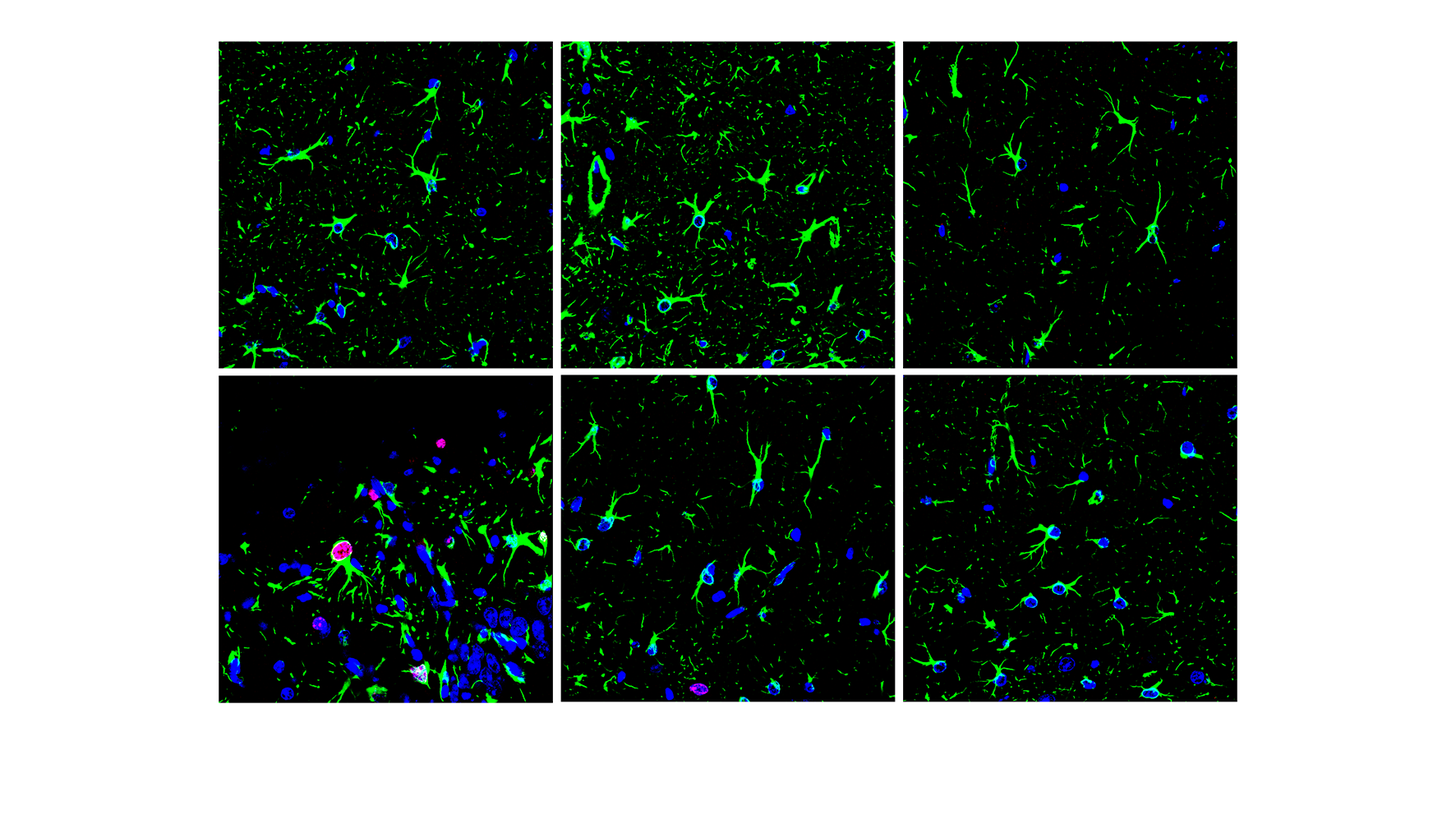

Supplement: Supplementary file 6 [file Image_4.TIF]

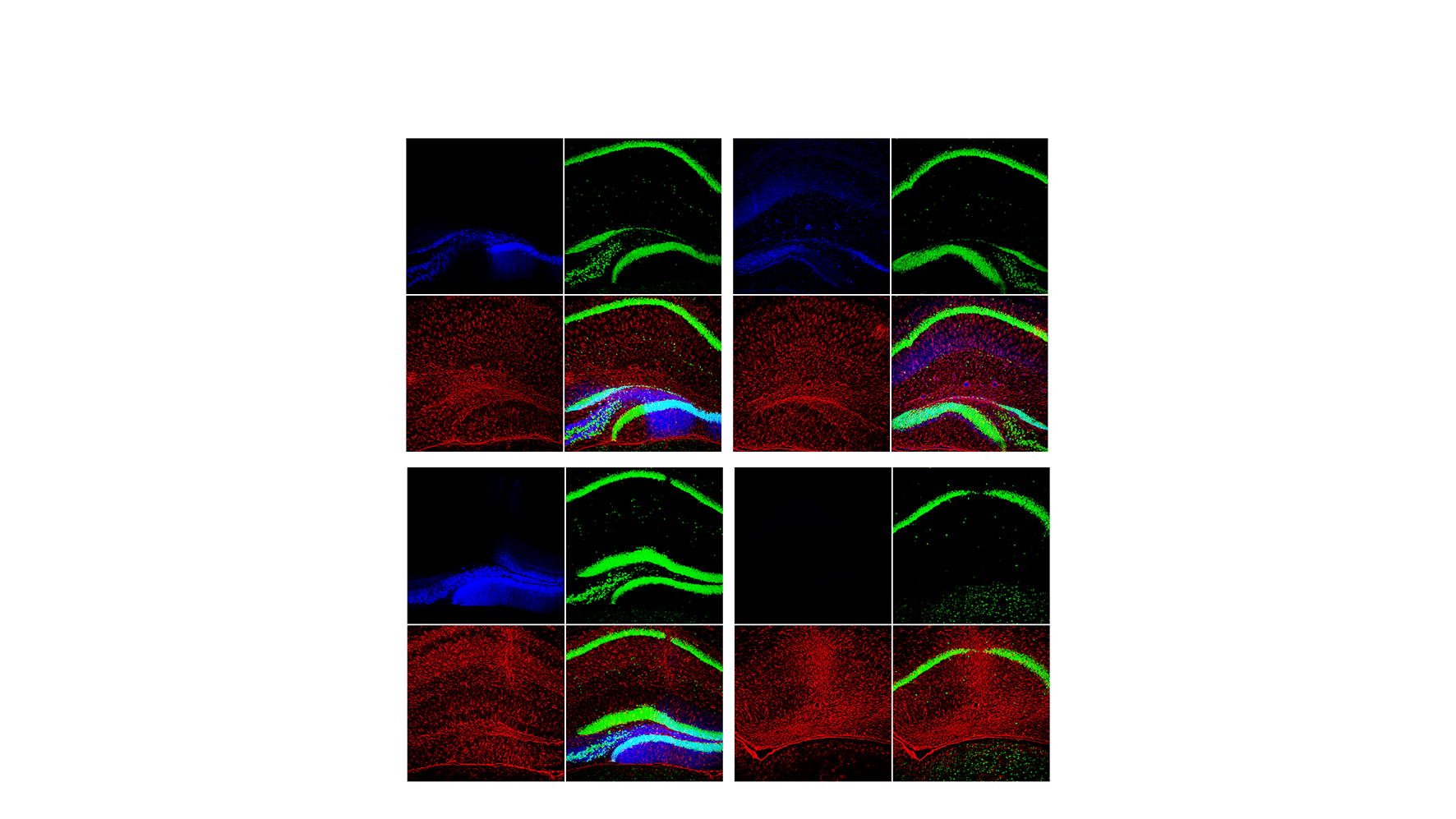

Supplement: Supplementary file 7 [file Image_5.TIF]

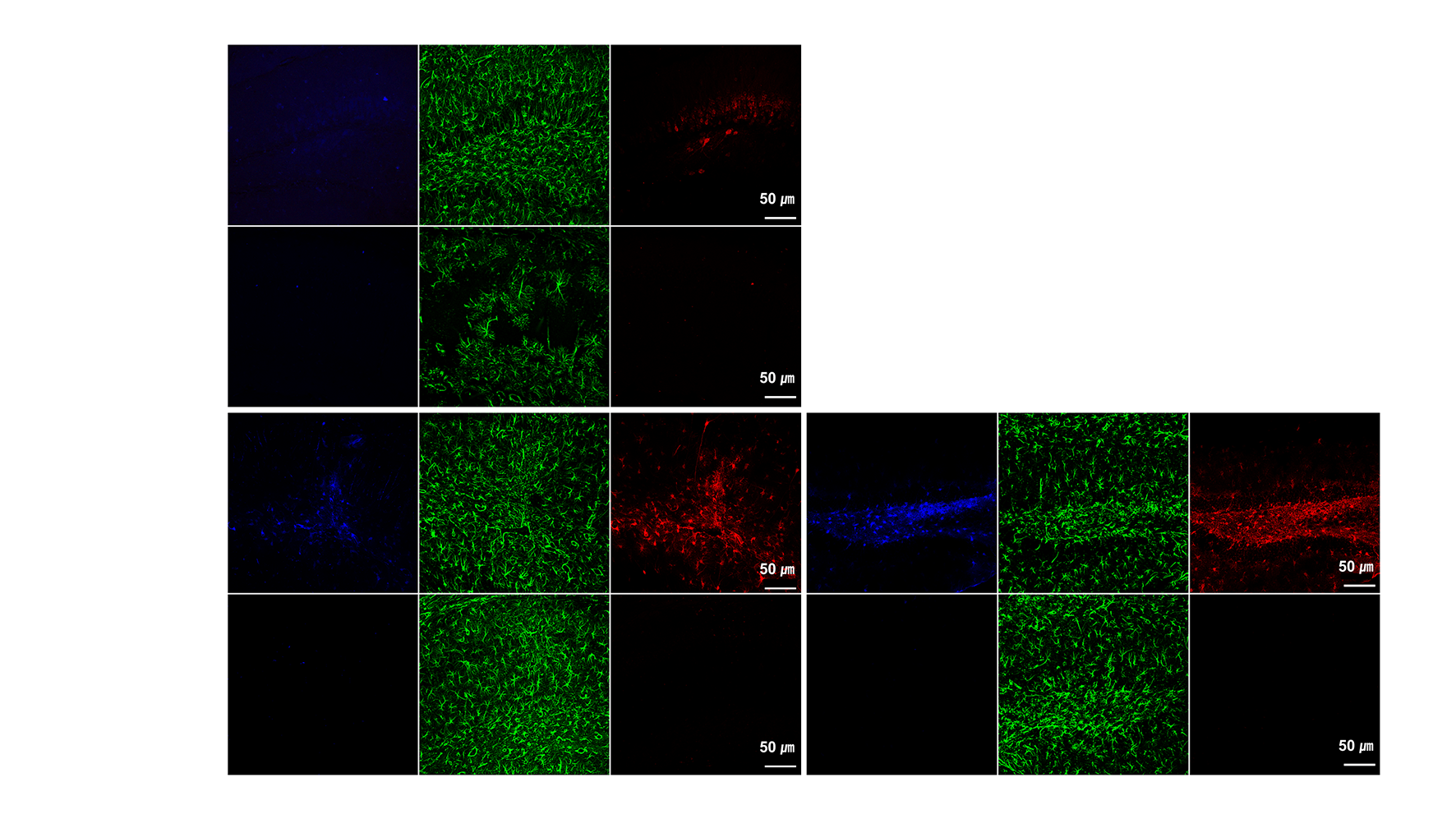

Supplement: Supplementary file 8 [file Image_6.TIF]

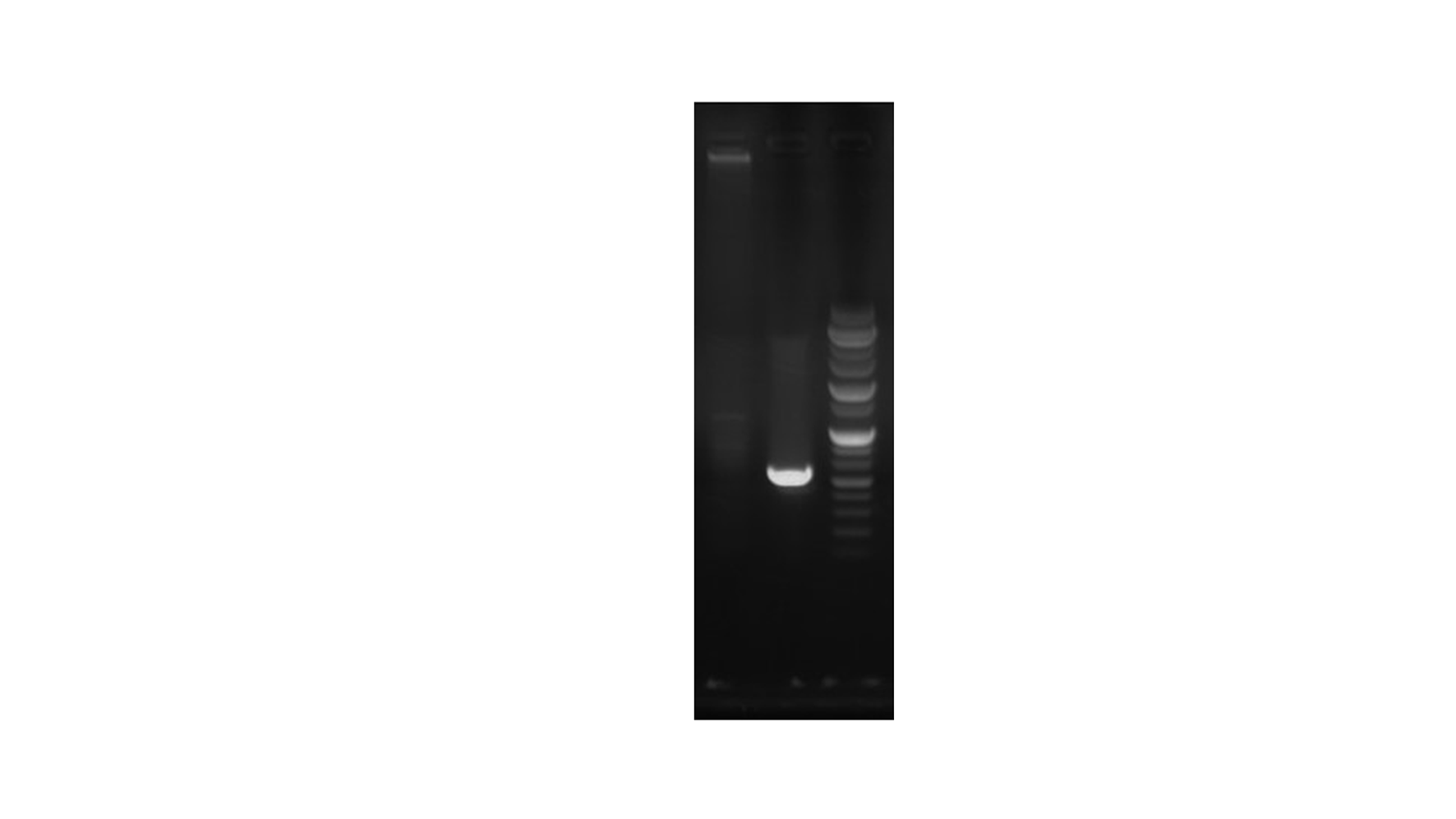

Supplement: Supplementary file 9 [file Image_7.TIF]

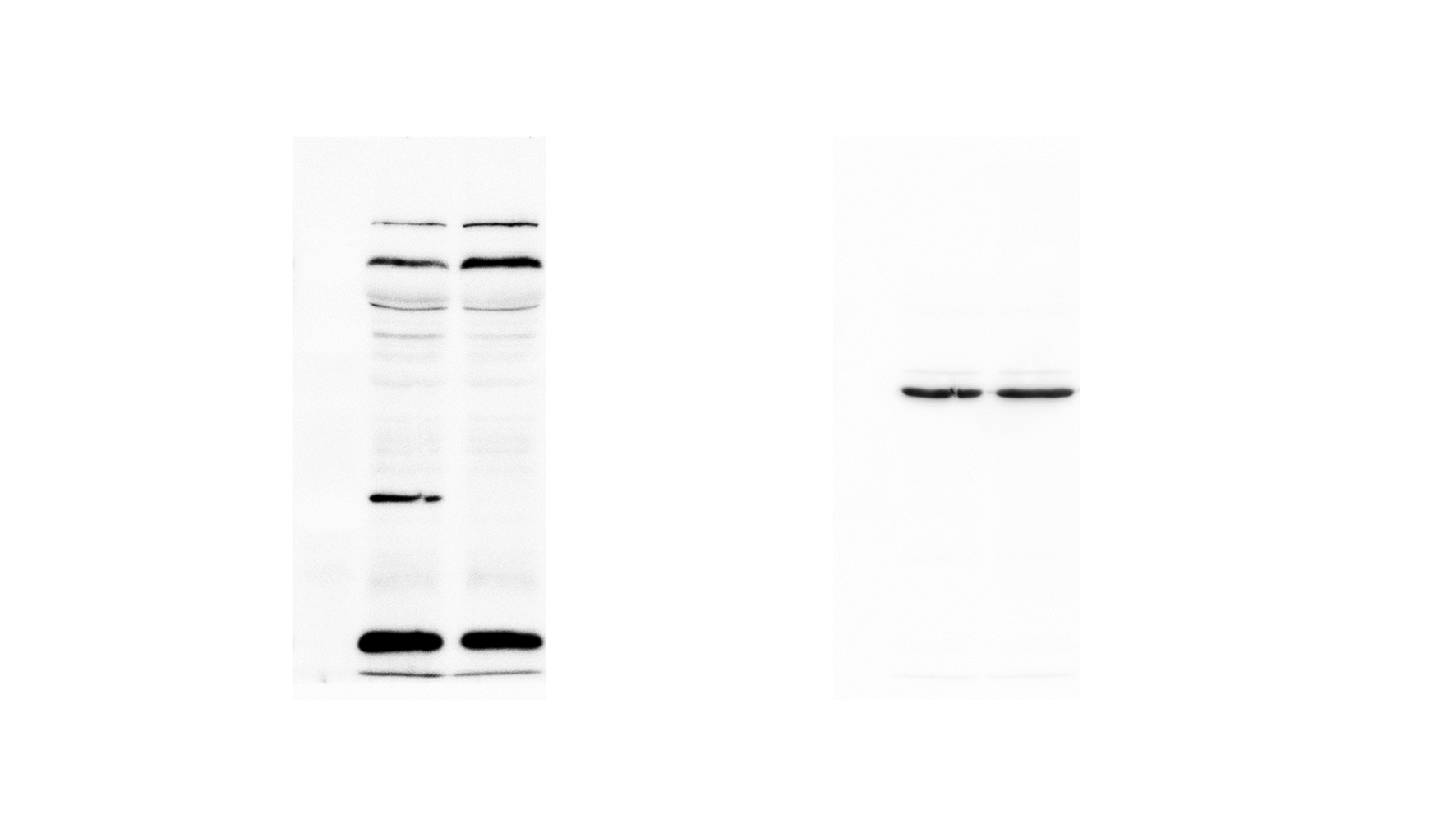

Supplement: Supplementary file 10 [file Image_8.TIF]

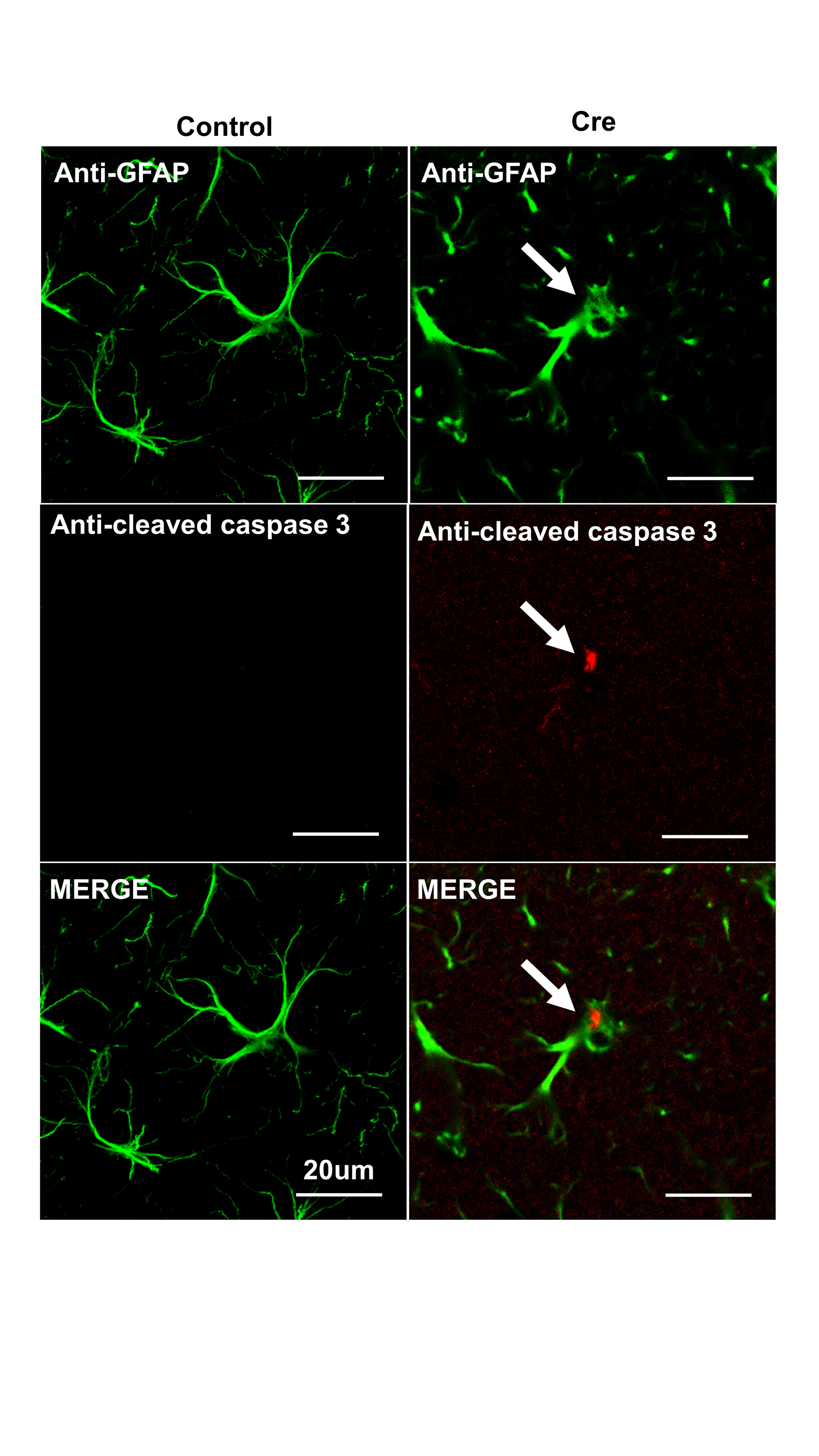

Supplement: Supplementary file 11 [file Image_9.TIF]

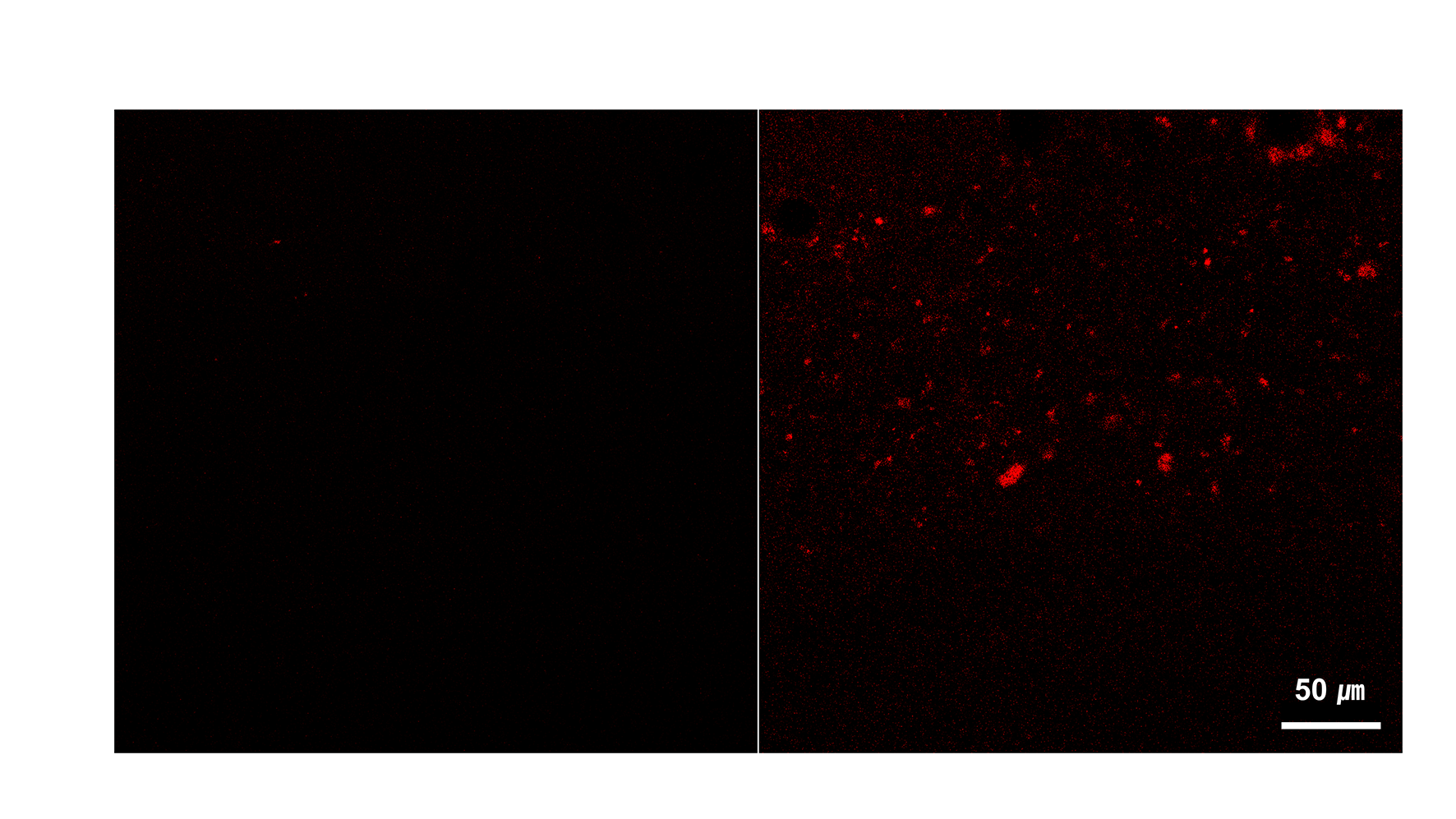

Supplement: Supplementary file 12 [file Image_10.TIF]
